# Hexapod robot motion planning investigation under the influence of multi-dimensional terrain features

**DOI:** 10.3389/fnbot.2025.1605938

**Published:** 2025-05-21

**Authors:** Chen Chen, Junbo Lin, Bo You, Jiayu Li, Biao Gao

**Affiliations:** ^1^The Key Laboratory of Intelligent Technology for Cutting and Manufacturing Ministry of Education, Harbin University of Science and Technology, Harbin, China; ^2^The Heilongjiang Provincial Key Laboratory of Complex Intelligent System and Integration, Harbin University of Science and Technology, Harbin, China

**Keywords:** hexapod robot, locomotion stability, multi-dimensional terrain features, path planning, free gait planning

## Abstract

To address the challenges arising from the coupled interactions between multi-dimensional terrain features—encompassing both geometric and physical properties of complex field environments—and the locomotion stability of hexapod robots, this paper presents a comprehensive motion planning framework incorporating multi-dimensional terrain information. The proposed methodology systematically extracts multi-dimensional geometric and physical terrain features from a multi-layered environmental map. Based on these features, a traversal cost map is synthesized, and an enhanced A* algorithm is developed that incorporates terrain traversal metrics to optimize path planning safety across complex field environments. Furthermore, the framework introduces a foothold cost map derived from multi-dimensional terrain data, coupled with a fault-tolerant free gait planning algorithm based on foothold cost evaluation. This approach enables dynamic gait modulation to enhance overall locomotion stability while maintaining safe trajectory planning. The efficacy of the proposed framework is validated through both simulation studies and physical experiments on a hexapod robotic platform. Experimental results demonstrate that, compared to conventional hexapod motion planning approaches, the proposed multi-dimensional terrain-aware planning framework significantly enhances both locomotion safety and stability across complex field environments.

## 1 Introduction

In recent years, autonomous field mobile robots have garnered increasing attention and remain at the forefront of research endeavors (Zghair and Al-Araji, 2021). Compared to wheeled and tracked robots, legged robots possess the capability to traverse discontinuous and uneven terrain by modulating their gait patterns, body postures, and foothold positions, while maintaining relative balance and stability (Biswal and Mohanty, 2021; Arrigoni et al., 2024). These characteristics enable them to effectively navigate obstacles and challenging environments. However, in unpredictable and unstructured field environments, the coupled variations in geometric features (such as ruggedness and steepness) and physical properties (such as soil compressibility and slipperiness) of the terrain pose significant challenges to autonomous motion planning for legged robots. Developing more rational and environmentally adaptive motion planning strategies for legged robots in complex field environments continues to be one of the primary research focuses among scholars in the field of legged robotics.

Current research in hexapod robot motion planning primarily encompasses two fundamental aspects: path planning (Wang et al., 2022; Zhang et al., 2023) and gait planning (Zhu et al., 2017; Yu et al., 2020). Path planning focuses on the macroscopic level, addressing the robot’s overall trajectory generation, while gait planning typically operates at a subordinate level, emphasizing the local-level planning of specific foothold sequences. These two aspects complement each other synergistically to achieve effective locomotion.

In the field of path planning research, scholars typically focus on planning the robot’s overall motion while considering both the robot’s locomotion capabilities and environmental information to enhance path safety and feasibility. The Boston Dynamics BigDog robot (Ding, 2015) utilizes an A* algorithm based on a 2D cost grid map to generate shortest collision-free paths, enabling it to navigate around major obstacles such as trees and boulders in forest environments. However, this approach only considers terrain geometric features and optimizes for path distance without addressing path safety. The HyQ robot (Arain et al., 2013; Winkler et al., 2015) employs an A* algorithm with cost mapping to plan feasible paths in unstructured terrain. This path planning methodology evaluates traversability and risk levels based on obstacle geometric features to generate paths optimizing both distance and traversability. However, the approach only considers terrain geometric features while neglecting physical properties, resulting in suboptimal safety considerations.

In the domain of gait planning research, scholars primarily focus on how robots can enhance their adaptability to external terrain variations through optimal combinations of inter-leg motion sequences (Long et al., 2019; Cai et al., 2021; You et al., 2021). Cheah et al. (2018) investigated the impact of ground height variations on robot gait, utilizing 2.5D elevation maps to set robot foothold heights and improve locomotion stability, but they did not analyze the influence of terrain properties on robot foot placement. Wang et al. (2023) conceptualized the unstructured terrain as random stepping stones, employing deep reinforcement learning algorithms to plan foothold positions for a hexapod robot in the planar stepping stone environment. While this enhanced the robot’s mobility in unstructured environments, their binary classification of foothold regions as either suitable or unsuitable failed to quantitatively analyze the degree of terrain influence on robot foot placement.

Comprehensive analysis of current hexapod robot motion planning research reveals that in path planning, scholars predominantly consider only the influence of geometric terrain feature variations while neglecting the potential impact of physical terrain properties on path safety. However, in actual field environments, variations in geometric features and physical properties typically have coupled effects on overall robot performance. Planning paths solely based on geometric features significantly limits the practical applicability of hexapod robots in field environments. Simultaneously, in robot gait planning research, scholars often neglect the impact of multi-dimensional terrain features on robot foot placement. Some researchers implement binary classification of foothold regions, but without quantitative analysis of terrain influence on foot placement, which also constrains the practical field application of hexapod robots.

Therefore, addressing these challenges, a hexapod robot motion planning methodology under the influence of multi-dimensional terrain features is proposed. The approach extracts multi-dimensional geometric features and physical properties through multi-level mapping. Based on this, a traversability cost map is constructed, and the traditional A* algorithm is enhanced based on traversability costs to improve path safety across complex field terrain. Furthermore, a foothold cost map is developed using multi-dimensional terrain information, and a free gait planning method based on foothold cost is proposed, which further enhances overall robot motion safety and stability through gait adjustment under the planned safe motion path.

## 2 Map construction and multi-dimensional terrain feature analysis

### 2.1 Multi-layer map construction

**Figure 1:**
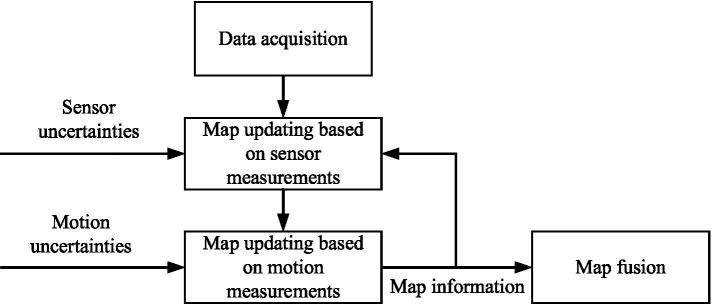
The 2.5D elevation map construction process.

The fundamental information of the environment is its geometric features. Therefore, a 2.5D geometric map of the terrain is constructed. During the mapping process, due to sensor noise and the inherent uncertainty in robot motion, these uncertainties must be considered. Referring to literature (Fankhauser et al., 2018), a 2.5D geometric map with uncertainty measurements is constructed. The mapping process is divided into three steps: first, updating the map based on depth information and sensor noise. Second, updating the map according to robot motion and position uncertainty information. Third, performing map fusion based on the height and covariance matrix information. The specific map construction process is shown in Figure 1.

The safety and stability of robots traversing complex outdoor environments are closely related to the geometric features and physical properties of the terrain. The current environment modeling methods only establish the geometric features of the ground and cannot identify hazardous terrains such as loose sand or water holes. Therefore, it is necessary to construct a semantic layer map containing terrain categories to enrich the environment information.

**Figure 2:**
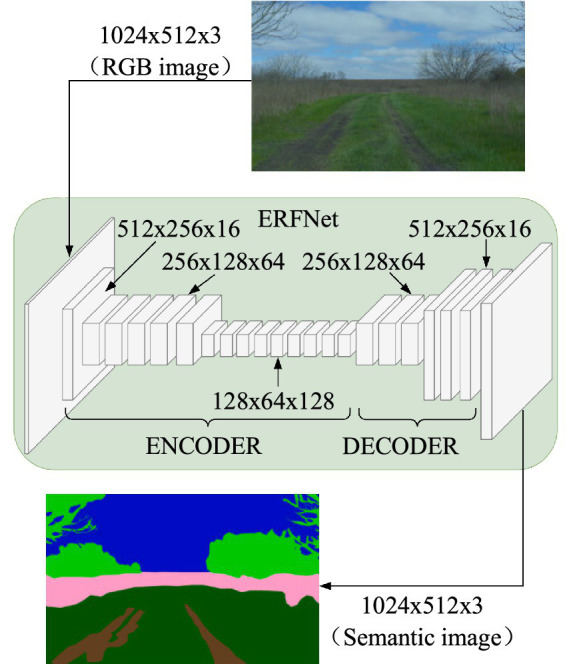
Schematic diagram of ERFNet network architecture.

Semantic segmentation assigns a label from a set of predefined terrain categories to each pixel in the input image, enabling robots to determine the categories of surrounding terrain. We employs the ERFNet network architecture proposed by Romera et al. (2018) as the neural network architecture for the semantic segmentation module, as illustrated in Figure 2.

**Figure 3:**
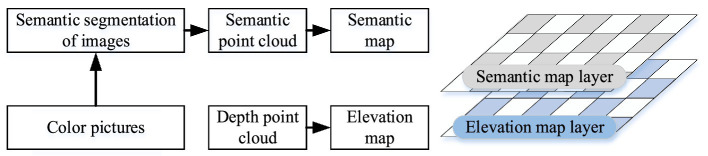
Schematic diagram of multi-layer map model.

Through semantic segmentation models, a 2D ground surface segmentation image is obtained in the camera coordinate system. First, the semantic image is converted into a semantic point cloud. Then, based on the transformation relationship between the camera and map coordinate systems, the semantic point cloud is projected onto the map coordinate system. Finally, a multi-layer map model containing ground elevation and semantic information is generated, as illustrated in Figure 3.

### 2.2 Multi-dimensional terrain information analysis

Although the multi-layer map contains rich environmental information, it cannot quantitatively characterize the environment’s impact on motion planning. To enable robots to better understand how their environment may affect their movement, further analysis of the robot’s multi-dimensional ground surface information should be conducted to obtain quantitative representations for evaluating the ground surface conditions where the robot operates.

**Figure 4:**
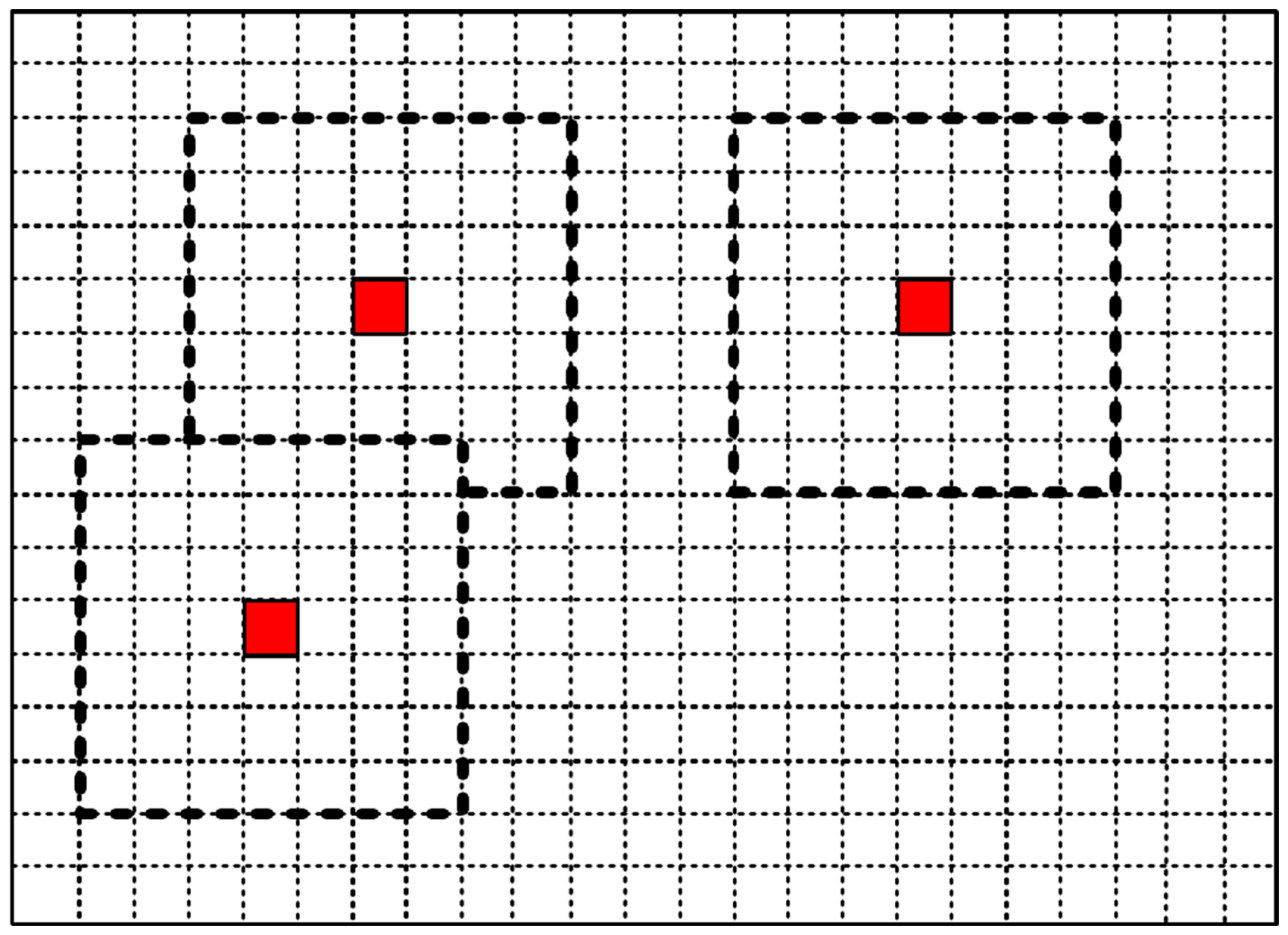
Schematic diagram of sliding window.

A sliding window method is adopted to analyze ground surface information, with adjustable window sizes according to requirements. When calculating traversal costs for path planning, the robot is considered as a whole, and the sliding window size should approximate the robot body dimensions. When calculating foothold costs for gait planning, the window size should be close to the foot sole dimensions. To analyze grid information based on adjacent grids, the window should contain at least 9 grids. Figure 4 illustrates the sliding window, where the red grid represents the currently analyzed grid, and the dashed box indicates the established sliding window.

During locomotion, hexapod robots cannot traverse areas where the slope exceeds their climbing ability. When facing a step obstacle that exceeds the robot’s step height, the robot cannot traverse it. Hexapod robots require sufficient workspace for leg lifting and placement; excessive step height greatly increases the probability of collision with step edges, significantly affecting the robot’s stability. Excessive ground undulation causes substantial body fluctuation during locomotion, affecting stability. Different terrain types have varying friction and softness characteristics. Friction plays a decisive role in whether the robot slips during locomotion—for instance, robots are more likely to slip on muddy ground compared to grassy surfaces. Softness determines whether the robot sinks—robots are more prone to sinking in sandy terrain compared to soil. Therefore, analyzing ground slope, gradient, surface undulation, friction coefficient, and substrate softness is vital.

Assuming the window contains *N* grid points, the height information $(x_{i},y_{i},h_{i}),i=1,2,\cdots N$ of each grid is obtained from the elevation map. Using the least squares method, the plane equation fitted from *N* grid data is depicted in equation 1:

(1) z=Ax+By+C

Based on this plane equation, the normal vector $\vec{n}$ can be calculated as shown in equation 2:

(2) $$\vec{n}=\frac{\left(-A,-B,1\right)}{-A,,-B,,1}$$

Setting the reference plane as the horizontal plane with normal vector ${\vec{n}}_{0}=(0,0,1)$ , the slope of the fitted plane can be calculated from the normal vectors as shown in equation 3:

(3) $$\left\{ \begin{array}{l} \cos\theta =\frac{\vec{n}{\vec{n}}_{0}}{|\overset{⇀|}{n}|{\vec{n}}_{0}|}=\frac{1}{\sqrt{A^{2}+B^{2}+1}}\\ \theta =\arccos\frac{1}{\sqrt{A^{2}+B^{2}+1}} \end{array}\right.$$

The gradient is a macroscopic factor representing the surface gradient’s degree. It is computed as the difference between the raster’s maximum and minimum elevation values within the sliding window. By traversing the height value of each raster within the sliding window and recording the maximum and minimum elevation values as $H_{\max}$ and $H_{\min}$ , respectively, the gradient can be determined as *h* shown in equation 4:

(4) $$h=H_{\max}-H_{\min}$$

Undulation is a term used to describe whether the surface is flat and is typically expressed as the standard deviation of elevation values within the analysis window. *r* denotes the undulation as shown in equation 5:

(5) $$r=\sqrt{\frac{1}{N}\sum_{i=1}^{N}(h_{i}-h_{u})}$$

Ewen et al. (2022) established friction values (expressed as $\tilde{f}$ ) and looseness values (expressed as $\tilde{m}$ ) for 10 different terrains, namely $\tilde{f},\tilde{m}\in [0,1]$ . The specific details can be found in Table 1.

**Table 1:** Terrain friction and softness.

Surface	Friction $\tilde{f}$	Looseness $\tilde{m}$
Invalid surface	1	1
Land	1	0.25
Grassland	0.7	0.4
Asphalt road	0.7	1
Stone Road	0.85	0
Snow	0.25	0.85
Sandy	0.8	1
Shrubs	0.55	0.5
Puddles	0.1	1
Mud	0.3	0.65

Based on the surface information in Table 1, the sliding window method is used to analyze the friction characteristics. The average friction value of the grid within the window is used as the friction value of the current analysis grid. The number of grids in the sliding window is *N*, and the friction degree of each grid is ${\tilde{f}}_{i}$ . The calculation for the looseness is depicted in equation 6:

(6) $$f=\frac{1}{N}\sum_{i=1}^{N}{\tilde{f}}_{i}$$

The calculation method for determining looseness properties is analogous to that of friction analysis and can be expressed as equation 7:

(7) $$m=\frac{1}{N}\sum_{i=1}^{N}{\tilde{m}}_{i}$$

## 3 Motion planning of the hexapod robot

### 3.1 Path planning based on multi-dimensional terrain information

#### 3.1.1 Traversal cost analysis based on multi-dimensional terrain information

**Figure 5:**
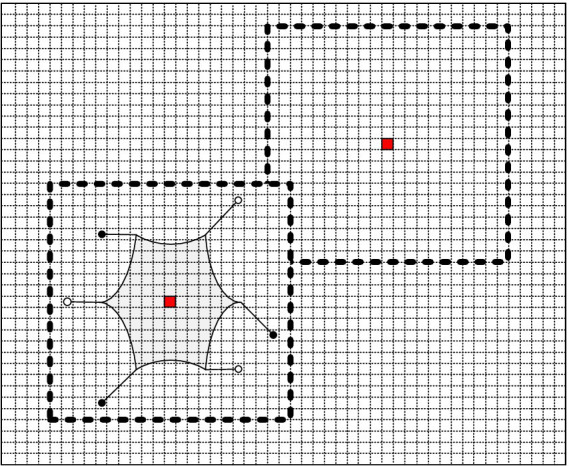
The sliding window of crossing cost estimation.

When planning a robot’s path, it is essential to consider the robot as a complete entity and analyze how terrain characteristics affect its traversability. The sliding window size should be configured to approximate the robot’s two-dimensional footprint. Given that the robot’s maximum extension occupies a rectangular area with a 20 mm grid size, the sliding window should contain a corresponding number of grid cells, a rectangle with a grid size of 20 mm has a maximum extension size of for the robot, so the number of grids in the sliding window is taken as N=21×21 , as shown in Figure 5.

If the terrain is excessively soft, the hexapod robot may experience sinking, hindering its traversal. Additionally, steep inclines surpassing the robot’s limit for climbing may also impair its ability to move forward. Likewise, excessive undulation on the surface may cause the robot’s body fluctuations. Furthermore, obstacles with step heights beyond the robot’s limit may pose traversal difficulties. Thus, the traversal cost $t_{p}$ of the map can be computed by considering the terrain softness, slope, and gradient information. The corresponding formula is depicted in equation 8.

(8) $$t_{p}=\{\begin{array}{l} \;\;1\;\;(\text{if}\;\;\theta >\theta_{\text{crit}}\;\;\text{or}\;\;h>h_{\text{crit}}\;\;\text{or}\;\;r>r_{\text{crit}}\;\;\text{or}\;\;m<m_{\text{crit}})\\ w_{1}\frac{\theta }{\theta_{\text{crit}}}+w_{2}\frac{h}{h_{\text{crit}}}+w_{3}\frac{r}{r_{\text{crit}}}+w_{4}(1-\frac{m}{m_{\text{crit}}})\;\;(\text{else}) \end{array}$$

where $w_{1},w_{2},w_{3},w_{4}$ represent weights assigned to the slope, gradient, undulation, and softness factors. These weights can be adjusted to meet the specific needs of the engineering task at hand. This study assigns all aspects equal importance, with $w_{1},w_{2},w_{3},w_{4}$ set at 0.25 for each. $\theta_{\text{crit}}$ , $h_{\text{crit}}$ , $r_{\text{crit}}$ , and $m_{\text{crit}}$ represent slope, gradient, undulation, and looseness thresholds. These thresholds must be set based on the actual climbing ability, step height, flatness goal, and robot weight and foot area, thus ensuring optimal robot traversal.

#### 3.1.2 Improved A* algorithm based on traversal cost

To address loose terrain surfaces like sand and puddles, we enhance the conventional A* algorithm by considering the terrain’s multi-dimensional geometric features and physical properties. This integration allows for analyzing the traversal cost on such surfaces. The traditional A* algorithm is shown in equation 9.

(9) f(n)=g(n)+h(n)

We have enhanced the conventional A* algorithm by incorporating the traversal cost factor, ultimately enabling the robot to locate the most cost-efficient route. The resulting, improved algorithm is displayed in equation 10.

(10) $$f(n_{\text{next}})=g(n_{\text{next}})+h(n_{\text{next}},e)+\text{cost}(n_{\text{next}})$$

Equation 11 governs the expressions of $g(n_{\text{next}})$ and $\text{cost}(n_{\text{next}})$ .

(11) $$\left\{ \begin{array}{l} g(n_{\text{next}})=g(s,n)+g(n,n_{\text{next}})\\ \text{cost}(n_{\text{next}})=\text{cost}(s,n)+t_{p}(x,y) \end{array}\right.$$

**Figure 6:**
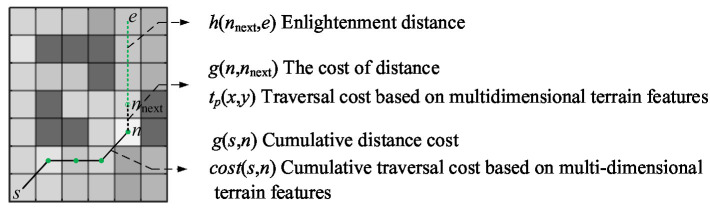
The schematic diagram of A* algorithm based on traversal cost.

The schematic diagram of the A* algorithm based on the traversal cost is shown in Figure 6.

The following variables are defined in equation form: $g(n,n_{\text{next}})$ represents the distance between path node *n* and the subsequent node $n_{\text{next}}$ . $t_{p}(x,y)$ represents the traversal cost based on multidimensional surface information for the raster in which $n_{\text{next}}$ nodes are situated. g(s,n) represents the cumulative path length from the origin (node *s*) to the current node *n*. cost(s,n) represents the incremental traversal cost based on multidimensional surface information from the source (node *s*) to the current node *n*. $h(n_{\text{next}},e)$ is the heuristic function used to identify the optimal path, where $h(n_{\text{next}},e)$ is determined by calculating the Chebyshev distance from node $n_{\text{next}}$ to the end point *e*.

**Figure 7:**
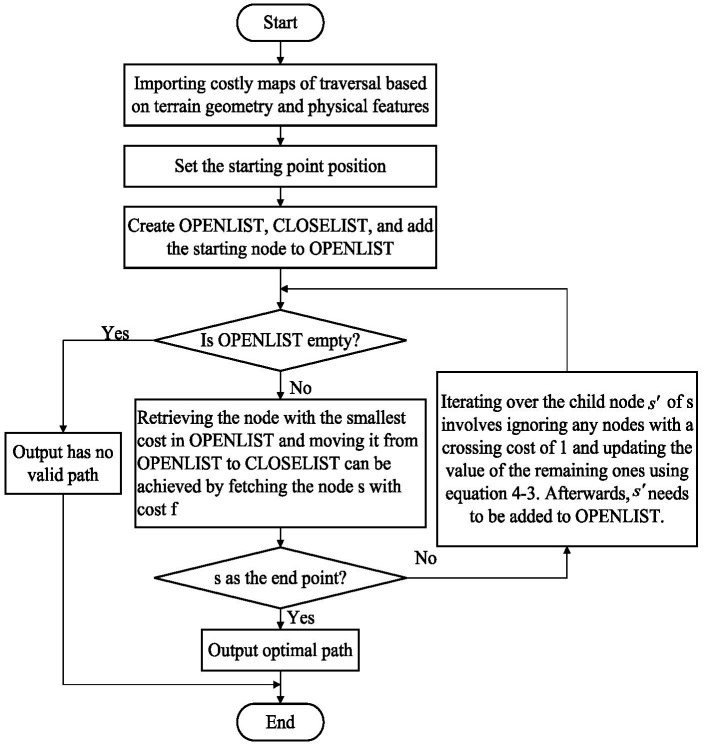
The overall workflow of the A* algorithm based on traversal cost.

The path search using the improved path planning algorithm with traversal cost involves organizing traversed nodes in ascending order by $g(n_{\text{next}})+h(n_{\text{next}},e)+k*\text{cost}(n_{\text{next}})$ within a CLOSELIST. In contrast, nodes yet to be traversed are stored in an OPENLIST. Figure 7 illustrates the algorithm’s workflow.

### 3.2 Gait planning based on multi-dimensional terrain information

#### 3.2.1 Foothold cost analysis using multi-dimensional terrain information

**Figure 8:**
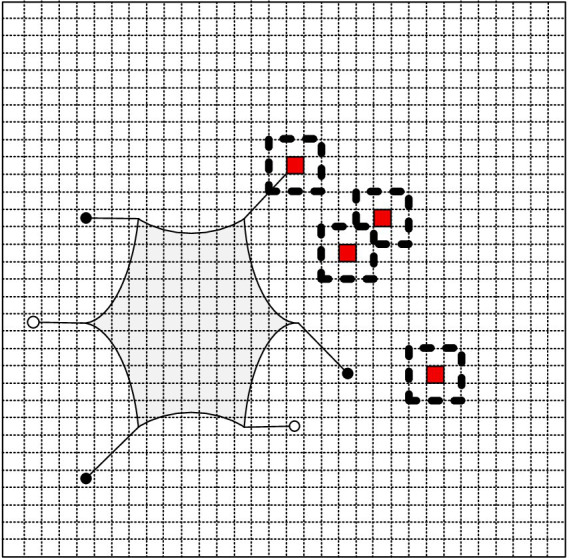
The sliding window of foothold cost estimation.

When performing gait planning for the robot, it is essential to consider the impact of the terrain surface on foot placement. To this end, the sliding window size is set to approximate the size of the robot’s foot, with the grid size set to a rectangular shape measuring 20 mm. Given that the robot’s foot measures approximately 15 mm × 15 mm, smaller than a single grid, a feature analysis using proximity grids requires a total of N=3×3 sliding window grids, as illustrated in Figure 8.

When a hexapod robot’s foot is landing, the ground surface it lands on can significantly impact its stability and safety. For example, a loose surface may cause the robot’s foot to sink, increasing the risk of tipping over. Significant gradient can also pose a challenge, as there is a high probability of the robot colliding with the step’s edge during the lifting and landing process, ultimately impacting the robot’s stability. Moreover, excessive undulation of the ground surface can cause the robot’s body to fluctuate, while insufficient friction can cause the robot’s foot to slip. Therefore, considering the surface’s gradient, friction, and looseness information, calculate the map’s foothold cost, as shown in equation 12.

(12) $$t_{d}=\{\begin{array}{l} \;\;1\;\;\;\;(\text{if}\;\;h>h_{\text{crit}}\;\;\text{or}\;\;f<f_{\text{crit}}\;\;\text{or}\;\;m<m_{\text{crit}})\\ w_{1}\frac{h}{h_{\text{crit}}}+w_{2}(1-\frac{f}{f_{\text{crit}}})+w_{3}(1-\frac{m}{m_{\text{crit}}})\;\;(\text{else}) \end{array}$$

The evaluation weights for gradient, friction, and looseness are denoted by $w_{1},w_{2},w_{3}$ in equation 12. All three factors are considered equally important in this paper. Thus, $w_{1},w_{2},w_{3}$ are set to 1/3. The gradient threshold, $h_{\text{crit}}$ , is determined based on the desired crash risk, while the friction threshold, $f_{\text{crit}}$ , reflects the actual plantar friction characteristics of the robot. Finally, the robot’s overall weight determines the looseness threshold, $m_{\text{crit}}$ . Calculate the footing cost of a raster by comparing the gradient, friction, and looseness feature values to their respective threshold values. If these values exceed the thresholds, the footing cost of the raster is evaluated as 1, thereby rendering the raster unsuitable for foothold.

#### 3.2.2 Free gait planning

**Figure 9:**
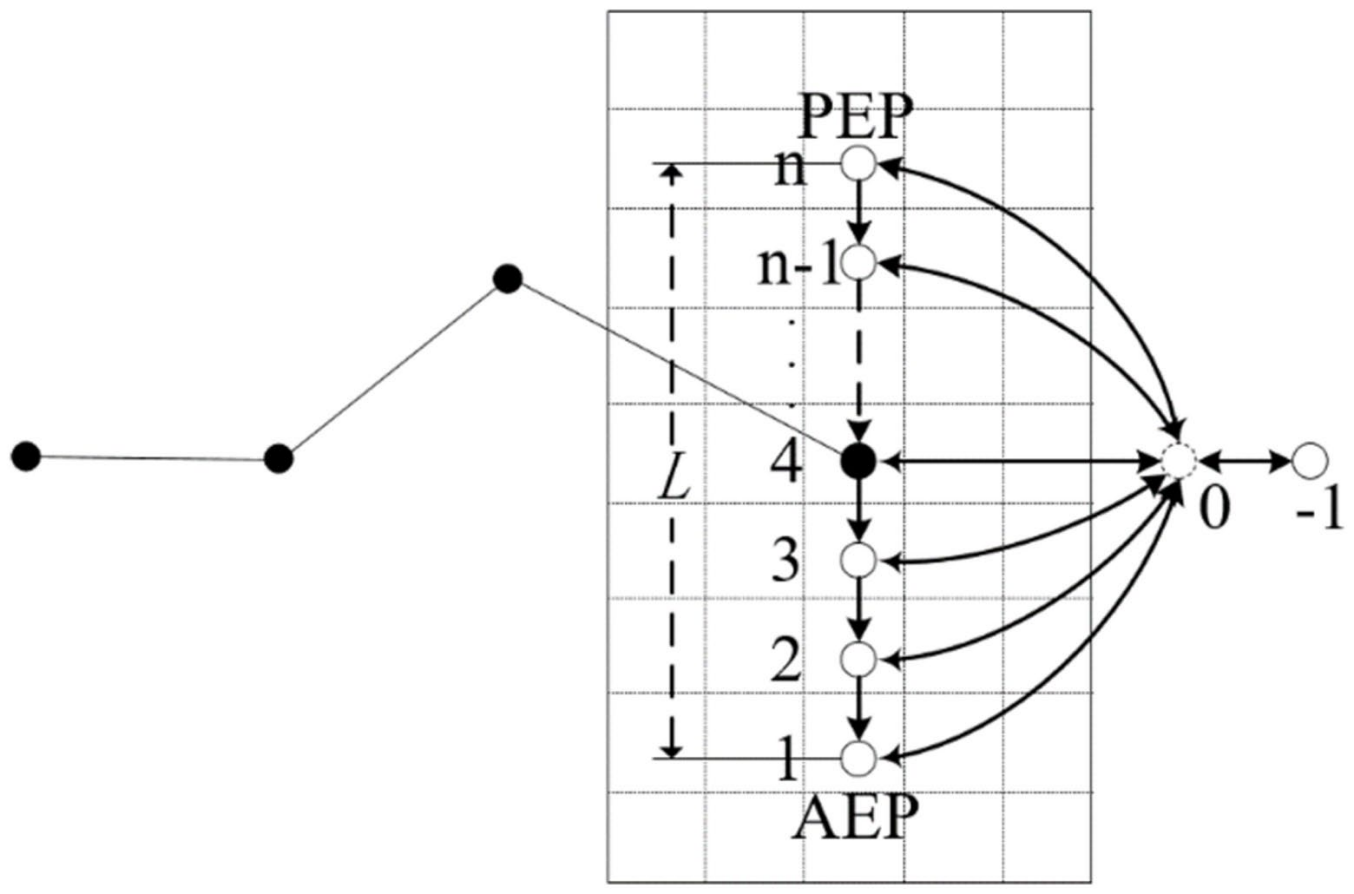
The discretization model of foot position state.

Conventional hexapod robot gaits typically employ a periodic rate with a fixed stride length, suitable for structured indoor environments. However, dynamic step length adjustment is required to enable a flexible selection of foothold points in obstacle areas when navigating complex and unstructured terrains. Therefore, based on the cost of the foothold map, an elastic-free gait is designed to meet the needs of robots walking in unstructured environments. Discretize the representation of the robot foot position, and the discretized model is shown in Figure 9.

Figure 9 illustrates the specific rules used for discretizing the position of the single-legged robot’s foothold, which are as follows:

1. During the leg’s support phase, n(n≥2,n∈Z) discrete equidistant points are inserted between the robot’s single-leg stride *L*. The foothold is located at the front limit point (AEP) as position *n*, the rear limit point (PEP) is position 1, and the discrete accuracy is determined based on the robot’s stride length *L* and grid size $L_{\text{cell}}$ , with $n=L/L_{\text{cell}}+1$ .
2. The end of the foot is assigned position 0 when the leg is in the swing phase with a foothold point presented.
3. The end of the foot is assigned position −1 when the leg is in the swing phase with no foothold point presented.
4. When the robot’s body needs to make a turn, one leg’s front and rear limit points require adjustment.

Transitions between foot-end positions follow the below rules:

1. Foothold positions can transition only from a more significant position point to a smaller one when the place ranges from 1 to *n*.
2. When the foot end position is 0, it can transition to either positions 1 to *n* or −1.
3. The foot end position can transition to positions −1 and 0 at −1.
4. All transitions occur within a single oscillation cycle.

Dynamic step length adjustment can be achieved by allowing the 0-position conversion to any position between 1 to *n* or −1. This feature enables the robot to traverse small, non-fillable areas safely. In the event of a non-fallible area too large for a single leg to have a fall point, the portion is identified as being in an erro state. It is subsequently controlled to hover at a specified position within a defined period, providing the gait with fault tolerance capabilities.

The robot’s position state is represented as the set of each leg state, denoted as $P_{k}=(p_{1}p_{1}\;p_{2}\;p_{3}\;p_{4}\;p_{5}\;p_{6})$ , with $p_{i}$ being the position of the i_th leg. *k* is the current oscillation period, and there are n+2 position states for a single portion of the hexapod robot, so there are ${\left(n+2\right)}^{6}$ robot position states, constituting a 6-dimensional robot position state space. The essence of gait planning is to plan a sequence of position states in the stable position state space to meet the robot motion requirements based on ground-based information, stability margin constraints, and transition rules between position states.

Assuming that the current position state $P_{n}$ of the hexapod robot is $\left(P_{1}\;P_{2}\;P_{3}\;P_{4}\;P_{5}\;P_{6}\right)$ , the grid size is $L_{\text{cell}}$ , the period of the robot’s rhythmic motion is $T_{p}$ , and the robot body moves at a speed $v(L_{\text{cell}}/T_{p})$ , the free gait planning is divided into the following four steps:

The first step is to preprocess the current position status $P_{n}$ . To identify the hidden position 0 in the current position state, the position where the next cycle will be in the swing phase, preprocess the current position state $P_{n}$ according to equation 13.

(13) $$p_{i}^{1}=\{\begin{array}{l} p_{i}\;\;\;p_{i}\geq v\\ 0\;\;\;0<p_{i}<v\\ 0\;\;p_{i}=\text{erro} \end{array}$$

The second step is to solve for the optional foothold positions. According to the current status and the information of the foothold cost grid, we can get the foothold position $p_{\mathrm{oi}}=\{(x_{1},y_{1}),(x_{2},y_{2}),\cdots (x_{n},y_{n})\}$ which the robot can reach in the next cycle, where if $\left(x_{L},y_{L}\right)$ is 1, that is, the obstacle grid. The part of the i_th footstep is *L*, and the set $L_{i}=\{l_{1},l_{2},l_{3},\cdots l_{m}\}$ saves all the optional foothold positions. When the grid in $p_{\mathrm{oi}}$ is fully occupied, there is no optional footing point for the i_th foot, denoted as $p_{\mathrm{oi}}=\text{erro}$ .

The next position state is solved in the third step according to the transition rule between position states. The possible position states of the robot for the next oscillation cycle are $P_{k+1}^{1}=(p_{1}^{2}\;p_{6}^{2}\;p_{3}^{2}\;p_{4}^{2}\;p_{5}^{2}\;p_{6}^{2})$ , where $p_{i}^{2}$ is determined as equation 14:

(14) $$p_{i}^{2}=\{\begin{array}{l} p_{i}^{1}-\nu \;\;\;\;p_{i}^{1}\neq 0\\ \text{random}(L_{i})\;\;\;\;p_{i}^{1}=0,p_{\mathrm{oi}}\neq \text{erro}\\ \text{erro}\;\;\;\;p_{i}^{1}=0,p_{\mathrm{oi}}=\text{erro} \end{array}$$

$$\text{The corresponding steady state}S_{n+1}^{1}=(s_{1}^{1}\;s_{2}^{1}\;s_{3}^{1}\;s_{4}^{1}\;s_{5}^{1}\;s_{6}^{1})$$

is depicted in equation 15:

(15) $$s_{i}^{1}=\{\begin{array}{l} 0\;\;\;p_{i}^{1}=0\\ 0\;\;\;p_{i}^{1}=\text{erro}\\ 1\;\;\;p_{i}^{1}\neq 0,p_{i}^{1}\neq \text{erro} \end{array}$$

The fourth step is stability determination. If position 0 occurs during the position transition, i.e., a leg of the robot is converted from the swing phase to the support phase, the value of $p_{i}^{2}$ may put the hexapod robot in an unstable state, and the precarious position state is divided into direct and indirect dangerous conditions. When the robot’s position state is (000000) , all six legs are in the swing state, and it is evident that the robot is unstable, and the state is directly dangerous. When the robot position state is (666666) , the robot is stable in the current cycle. After a certain period, the position state must be converted to a directly unstable state (000000) because the robot moves at the same speed as each foot when moving straight. Both direct and indirect instability states cannot occur during the gait planning of a hexapod robot.

Stability determination is performed for the position state corresponding to $p_{i}^{2}$ . The stability margin $\mathrm{SM}=\min\{s_{\mathrm{mf}},s_{\mathrm{mr}},s_{\mathrm{ms}}\}$ can be found according to equation 16 and equation 17.

(16) $$\left\{ \begin{array}{l} s_{\mathrm{mf}}=\max\{(s_{1}s_{6})\frac{y_{1}+y_{6}}{2},(s_{1}s_{5})\frac{(y_{1}+y_{5})X_{1}}{2(X_{1}+X_{2})},\\ \;(s_{2}s_{6})\frac{(y_{2}+y_{6})X_{1}}{2(X_{1}+X_{2})},(s_{2}s_{5})\frac{y_{2}+y_{5}}{2}\}\\ s_{\mathrm{mr}}=-\max\{(s_{2}s_{5})\frac{y_{2}+y_{5}}{2},(s_{2}s_{4})\frac{(y_{2}+y_{4})X_{1}}{2(X_{1}+X_{2})},\\ \;(s_{3}s_{5})\frac{(y_{3}+y_{5})X_{1}}{2(X_{1}+X_{2})},(s_{3}s_{4})\frac{y_{3}+y_{4}}{2}\}\\ s_{\mathrm{mr}}=\max\{s_{2}s_{5}X_{1},\max\{s_{1},s_{2},s_{3}\}\max\{s_{4},s_{5},s_{6}\}X_{2}\} \end{array}\right.$$

where

(17) $$\left\{ \begin{array}{l} y_{1}=J-\frac{L}{2}+\frac{(p_{1}-1)L}{n-1}\\ y_{2}=-\frac{L}{2}+\frac{(p_{2}-1)L}{n-1}\\ y_{3}=-J-\frac{L}{2}+\frac{(p_{3}-1)L}{n-1}\\ y_{4}=-J-\frac{L}{2}+\frac{(p_{4}-1)L}{n-1}\\ y_{5}=-\frac{L}{2}+\frac{(p_{5}-1)L}{n-1}\\ y_{6}=J-\frac{L}{2}+\frac{(p_{6}-1)L}{n-1} \end{array}\right.$$

$X_{1}$ is the lateral distance from the center of the robot to legs 1, 3, 4, and 6 and is the lateral distance from the center of the robot to legs 2 and 5. If the position state $S_{k+1}^{1}$ corresponds to the stability margin $SM_{k+1}^{1}\leq 0$ , then $p_{i}^{2}$ is a direct unstable state. If $p_{i}^{2}$ satisfies equation 18, it is an indirectly dangerous state. If $p_{i}^{2}$ is one of the dangerous states, then $p_{i}^{2}$ needs to be re-taken.

(18) $$\left\{ \begin{array}{l} k=\frac{\min\{p_{1}^{2},p_{2}^{2},p_{3}^{2},p_{4}^{2},p_{5}^{2},p_{6}^{2}\}}{v}\\ SM_{n+k+1}\leq 0 \end{array}\right.$$

**Figure 10:**
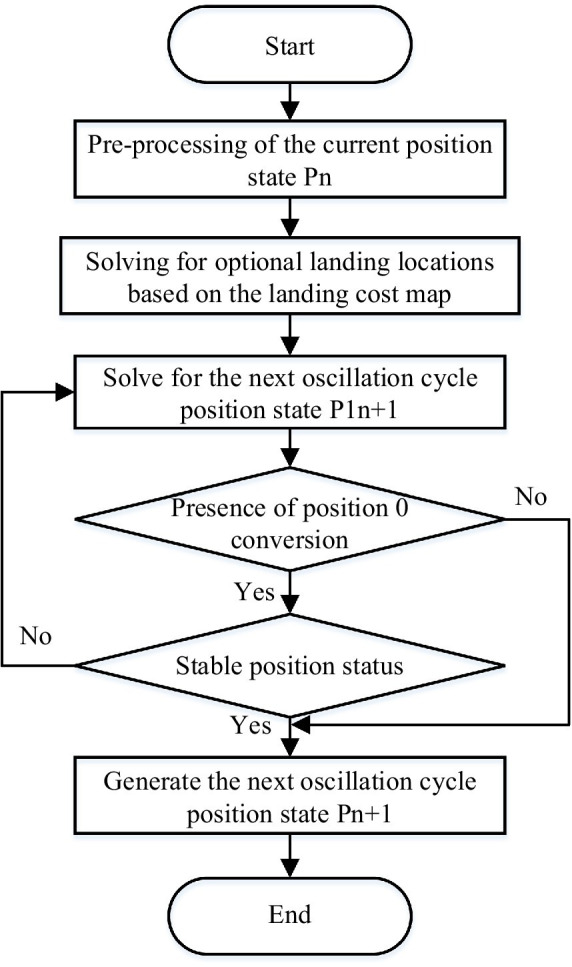
The algorithm flow of free gait planning.

After the above method, the next position state of the robot can be planned, and the algorithm flow chart is shown in Figure 10.

## 4 Experimental validation

**Figure 11:**
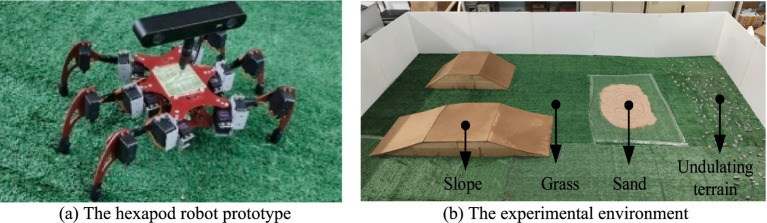
The hexapod robot prototype and the experimental environment. **(a)** The hexapod robot prototype. **(b)** The experimental environment.

To demonstrate the feasibility and effectiveness of the proposed hexapod motion planning investigation in improving the safety and stability of robot motion in complex environments, experiments on path and gait planning using real prototype platforms were conducted. Figure 11 displays the servo-driven hexapod robot with an aluminum body to reduce weight while ensuring greater stiffness. The initial posture size of the robot is L40 cm × W44 cm × H18 cm. The robot is equipped with a ZED2 depth camera and MPU6050 IMU sensor to provide environmental and robot attitude awareness. The experimental environment, shown in Figure 11b, is constructed with land, grass, sand, slopes, and obstacle-undulating terrain.

**Figure 12:**
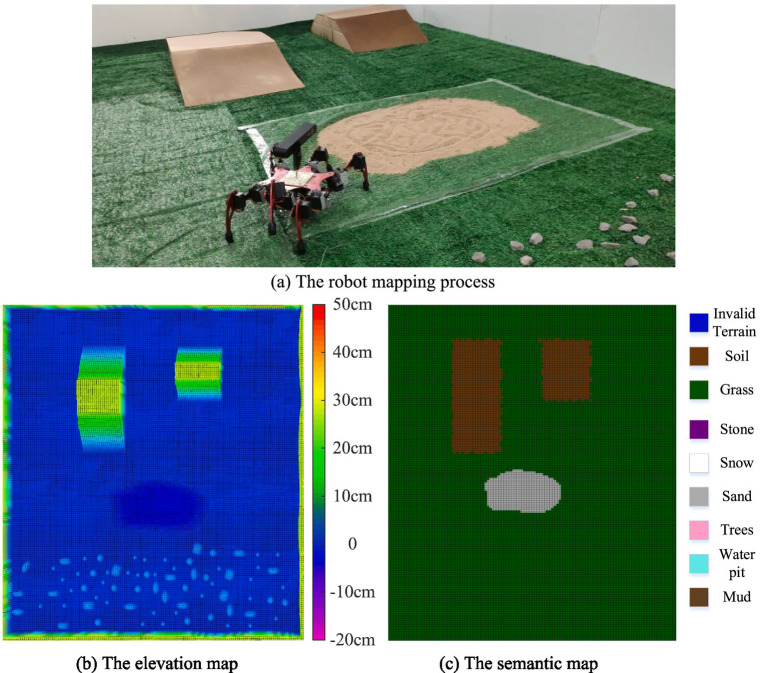
The robot mapping process and results. **(a)** The robot mapping process. **(b)** The elevation map. **(c)** The semantic map.

The environmental elevation map and semantic map were constructed using the parameters presented in Table 2. The mapping process and results are illustrated in Figure 12.

**Table 2:** Parameters of the map construction process.

Parameter	Value
Grid size (cm)	2
Motion speed	5
Map size (cm)	500 × 500
Terrain types	4

Figure 12b shows the established elevation map, where different colors correspond to different height values. Figure 12c illustrates the constructed semantic map, with different colors representing various terrain types. As can be observed from the mapping results, both the elevation map and semantic map are well constructed. However, the elevation map fails to identify hazardous terrains such as sand and water pits, the limitation that is effectively addressed by the implementation of the semantic map.

### 4.1 Experiments and analysis of robot path planning

To verify the effectiveness of the proposed robot path planning method, which considers traversability cost in enhancing robot motion safety in complex environments, robot path planning experiments were first conducted.

The traversal cost of the terrain was analyzed based on the elevation map and semantic map of the physical terrain. Path planning for the robot was performed based on the traversal cost map, with the path starting point set at (40, 9) and the endpoint at (89, 140). The parameters for constructing the traversability cost map were consistent with the parameters in Table 3.

**Table 3:** Traversal cost map construction parameters.

Parameter	Value
Sliding window size	21 × 21
Slope threshold	20°
Gradient threshold	8 cm
Undulation threshold	2 cm
Softness threshold	0.5

**Figure 13:**
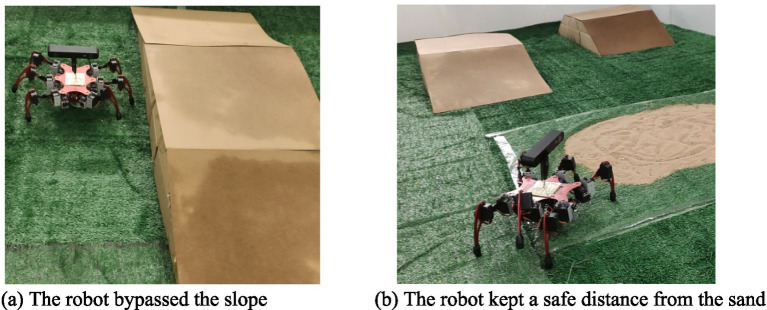
The path planning process of the robot. **(a)** The robot bypassed the slope. **(b)** The robot kept a safe distance from the sand.

**Figure 14:**
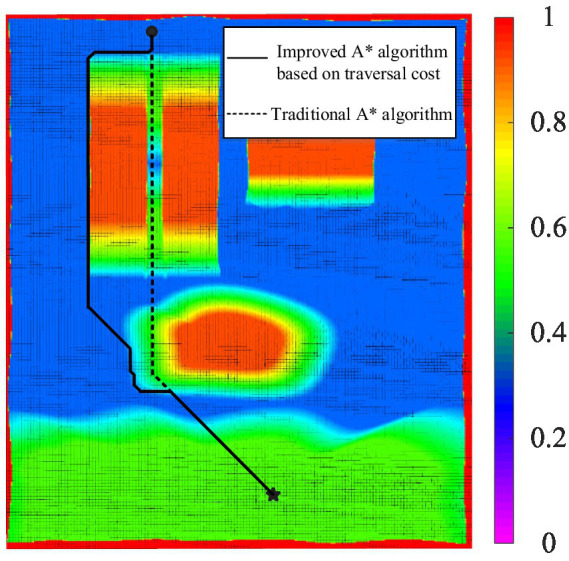
The traversal cost map and comparison of path planning results.

After creating the traversal cost map, path planning experiments using the improved A* algorithm based on traversal cost were conducted for the hexapod robot, and compared with path planning using the traditional A* algorithm. The path planning process is illustrated in Figure 13. The traversal cost map and the comparison of experimental results is shown in Figure 14.

From Figure 14, it can be observed that the robot can traverse the 15° slope on the left at a higher cost, while the 30° slope on the right exceeds the slope threshold of 20°, thus making it impassable for the robot. The large gradients on both sides of the slope are also impassable for the robot. The traversal cost for soft sandy terrain is 1, indicating that the robot cannot traverse it. The above analysis demonstrates that the traversal cost map considers not only the geometric characteristics of the terrain but also its physical properties. Furthermore, the areas surrounding slopes and sandy terrain also have high traversal cost, indicating that setting the sliding window size based on the robot’s dimensions for terrain feature analysis results in a traversal cost map that accounts for the impact of the robot’s size on terrain traversal, thereby allowing the robot to be treated as a point mass in subsequent path planning.

According to Figure 14, the traditional A* algorithm selects a path that traverses the slope and passes close to dangerous sandy terrain, whereas the improved A* algorithm based on traversal cost selects a path that bypasses the slope and maintains a safe distance from hazardous sandy terrain. The path planned by the proposed path planning method is safer, further validating the effectiveness of the proposed path planning algorithm in improving the motion safety of the robot.

### 4.2 Experiments and analysis of robot free gait planning

**Figure 15:**
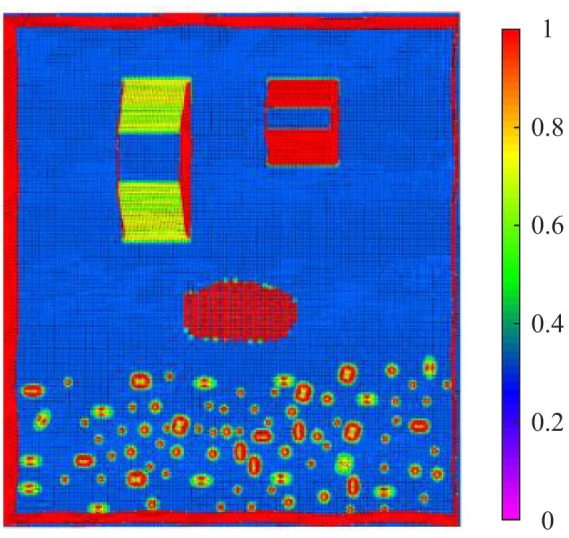
The foothold cost map.

To enable autonomous walking of the hexapod robot in complex environments, gait planning is required. The foothold cost map was constructed according to the parameters in Table 4, and Figure 15 shows the constructed foothold cost map.

**Table 4:** Foothold cost map construction parameters.

Parameter	Value
Sliding window size	3 × 3
Gradient threshold	3 cm
Friction threshold	0.65
Softness threshold	0.5

In Figure 15, purple represents a foothold cost of 0, while red represents a foothold cost of 1, indicating impassable terrain. As can be observed from the figure, the slope with a lesser gradient on the left has a lower foothold cost for the robot, whereas the slope with a greater gradient on the right has a higher foothold cost. The soft sandy terrain has a foothold cost of 1, and certain obstacle areas in the undulating terrain also have a foothold cost of 1, indicating that the robot cannot place its feet in these regions. Compared to the traversal cost map, the foothold cost map, due to its smaller analysis window, only considers the local terrain features of the foothold point, making it well-suited for gait planning.

To verify the effectiveness of the proposed free gait planning method in improving the motion performance of the robot, a straight-line path for the hexapod robot with the starting point at (20, 140) and the endpoint at (160, 140) was set. Comparative experiments were conducted between the free gait planning method proposed in this paper and the free gait planning method proposed in the author’s previous research (Zha et al., 2019). In the author’s previous research, a slope angle perception method based on plane fitting was proposed, along with a free gait planning approach for hexapod robots based on the perceived slope angle. In this method, the search space of available gaits is first narrowed by establishing a mapping relationship between the slope angle and the number of supporting legs. Then, by combining the robot’s stability constraint and leg motion space margin constraint to design a state search function, the method ultimately determines the gait options for the robot’s next step, enabling continuous gait planning. This approach does not utilize external sensors such as vision or LiDAR for environmental perception, thus the consideration of external environmental changes in this gait planning method is relatively limited.

**Figure 16:**
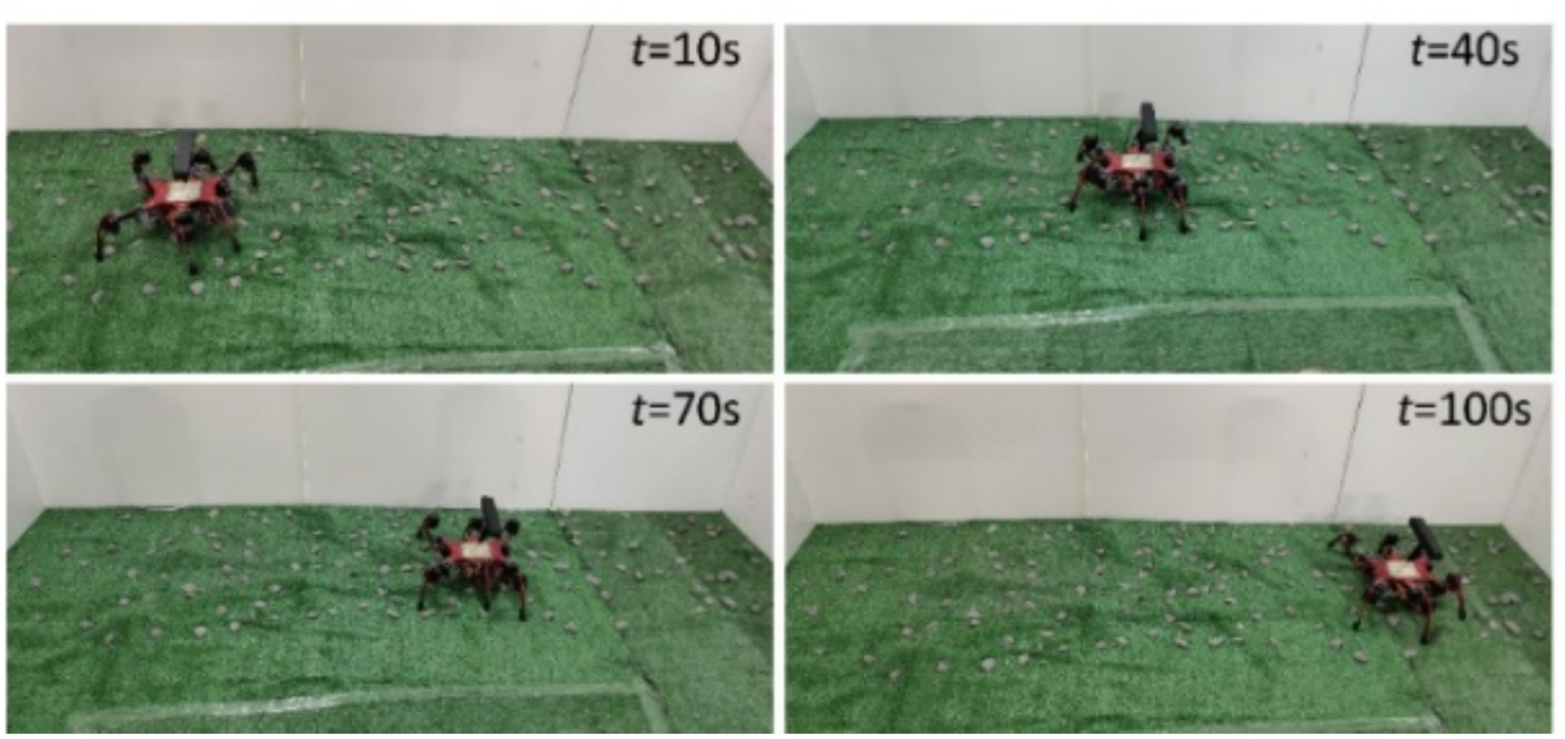
The walking process of the robot.

**Figure 17:**
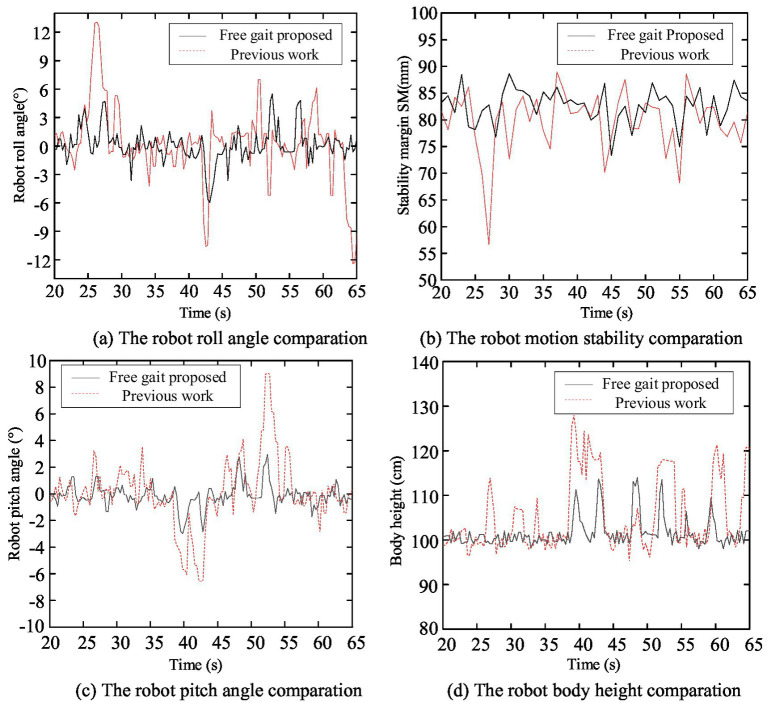
The robot body state curve comparation. **(a)** The robot roll angle comparation. **(b)** The robot motion stability comparation. **(c)** The robot pitch angle comparation. **(d)** The robot body height comparation.

Screenshots of the experimental process are shown in Figure 16, the robot body state curves are shown in Figure 17, and the robot body state data are presented in Table 5.

**Table 5:** The robot body state data.

Parameter	Proposed method	Previous work
Roll angle range (°)	−5.96 to 5.47	−12.44 to 13.07
Roll angle standard deviation (°)	1.76	3.95
Stability margin range (mm)	73.31–88.62	56.61–88.98
Average stability margin (mm)	82.66	79.77
Pitch angle range (°)	−2.98 to 2.97	−6.55 to 9.01
Pitch angle standard deviation (°)	0.89	2.57
Body height range (mm)	9.80–11.40	9.52–12.81
Body height standard deviation (mm)	0.33	0.81

From the data in Figure 17 and Table 5, it can be observed that the roll angle of the robot under the function of the free gait planning method proposed fluctuates minimally around 0°. Compared to the results of the author’s previous work, the standard deviation of fluctuation has been decreased by 55.4%, the average stability margin has been increased by 3.6%, the minimum stability margin has been improved by 29.5%, the pitch angle fluctuation has been reduced by 65.4%, and the body height fluctuation has been decreased by 59.3%.

In the free gait planning method proposed in the author’s previous research, the physical characteristics of the external terrain and their influence on the robot’s gait were not considered. Additionally, during the robot free gait planning process, only the variations in leg movement combinations were taken into account, without the ability to freely adjust parameters such as the robot’s stride length, resulting in limited freedom in the selection of foothold points. In the method proposed in this paper, the impact of multi-dimensional environment information on the robot’s foothold cost is considered and incorporated into the free gait planning method. Consequently, the robot can more flexibly select foothold points based on external terrain condition changes, better ensuring foothold safety and overall robot movement stability.

## 5 Conclusion

This paper analyzes the significant impact of terrain geometric features and physical properties on hexapod robot motion planning effectiveness, proposing a motion planning method that incorporates multi-dimensional terrain information. The method encompasses two primary levels: path planning and gait planning for hexapod robots that account for multi-dimensional terrain information influences. Specifically, multi-dimensional terrain information including slope, gradient, undulation, softness, and friction coefficient were analyzed using multi-layer maps. By comprehensively considering how multi-dimensional terrain information affects hexapod robot traversal safety, a terrain traversal cost map was constructed, and an improved A* algorithm based on traversal cost was proposed. Furthermore, by thoroughly examining the influence of multi-dimensional terrain information on hexapod robot foothold stability, a terrain foothold cost map was developed. Based on this foothold cost map, a free gait planning algorithm incorporating foothold cost was proposed, which further enhances the robot’s overall motion safety and stability through gait adjustments while ensuring safe path planning. Finally, through the implementation of physical experiments and subsequent data analysis, it was verified that the proposed hexapod robot motion planning method, which accounts for multi-dimensional terrain information, can effectively improve the safety and stability of hexapod robots traversing complex terrain compared to conventional motion planning methods.

## Data Availability

The original contributions presented in the study are included in the article/supplementary material, further inquiries can be directed to the corresponding author.
